# HDAC2-Specific Inhibitor Santacruzamate A Inhibits Mouse Preantral Folliculogenesis Through Granulosa Cell Apoptosis and Oocyte Meiosis Interference

**DOI:** 10.3390/ani16213336

**Published:** 2026-10-23

**Authors:** Fenglei Chen, Chengfei Wang, Tuba Latif Virk, Mengdie Pi, Yutong Chen, Qi Liu, Sheng Yang, Zhiyu Ma, Xianyu Xu

**Affiliations:** 1 College of Veterinary Medicine, Yangzhou University, Yangzhou 225009, China; 2 Jiangsu Co-Innovation Center for Prevention and Control of Important Animal Infectious Diseases and Zoonoses, Yangzhou 225009, China; 3 Jiangsu Interdisciplinary Center for Zoonoses and Biosafety, Yangzhou 225009, China

**Keywords:** preantral follicle, Histone deacetylase 2, Santacruzamate A, mouse

## Abstract

Histone deacetylase 2 (HDAC2)-mediated epigenetic modifications play a crucial role in oogenesis, yet the regulatory mechanisms of HDAC2 throughout the entire process of folliculogenesis remain elusive. In the present study, the localization and expression of HDAC2 were detected in mouse ovaries at distinct postnatal ages and after gonadotropin treatment. The effects and molecular mechanisms of Santacruzamate A (SCA), a selective HDAC2 inhibitor, on folliculogenesis were investigated via an in vitro 3D culture model of mouse preantral follicles. The results showed that HDAC2 was localized in the nuclei of both ovarian granulosa cells and oocytes. Its expression was gradually increased with ovarian development in mice aged from postnatal day 7 to 21, and significantly decreased after 12 h hCG treatment. In follicles cultured in vitro, HDAC2 expression peaked on day 4 of culture. SCA exerted stage-specific and dose-dependent regulatory effects on folliculogenesis. SCA significantly suppressed follicular growth, blocked antrum formation, and triggered follicular atresia. Mechanistically, SCA promoted ovarian granulosa cell apoptosis via increases in pro-apoptotic genes *BAX* and *Caspase-3* levels, and a decrease in anti-apoptotic gene *BCL-2* level. Furthermore, SCA disrupted the structural integrity of the follicular microfilament cytoskeleton, interfered with the levels of genes related to oocyte–granulosa cell communication including *CX37* and *GDF9*, caused abnormalities in oocyte spindle structure, chromosome alignment, mitochondrial distribution, and decreased mitochondrial membrane potential (ΔΨm). In conclusion, HDAC2 participates in the transcriptional regulation of mouse folliculogenesis through dynamically changing expression at distinct developmental stages. HDAC2 exerts stage-specific regulatory effects on follicular growth and oocyte maturation, and its mechanisms are associated with maintaining granulosa cell survival, ensuring cytoskeletal integrity, and preserving oocyte mitochondrial function. This study advances our understanding of epigenetic regulation of folliculogenesis and provides novel targets and an experimental basis for optimizing in vitro follicular culture systems and regulating female reproductive performance.

## Simple Summary

HDAC2-mediated epigenetic modifications play a crucial role in oogenesis, yet the regulatory mechanisms of HDAC2 throughout the entire process of folliculogenesis remain elusive. Studies focusing solely on isolated granulosa cells or oocytes cannot fully recapitulate the role of HDAC2 in folliculogenesis. Therefore, this study uses an in vitro 3D culture system of mouse preantral follicles to investigate the regulatory role of HDAC2 in folliculogenesis and its underlying molecular mechanisms. HDAC2 participates in the transcriptional regulation of mouse folliculogenesis through dynamically altered expression at distinct developmental stages. HDAC2 exerts stage-specific regulatory effects on follicular growth and oocyte maturation, and its mechanisms are associated with maintaining granulosa cell survival, ensuring cytoskeletal integrity, and preserving oocyte mitochondrial function.

## 1. Introduction

Efficient folliculogenesis is one of the core foundations for maintaining female fertility and the economic benefits of animal husbandry. Although livestock ovaries contain an abundance of undifferentiated preantral follicles, only a tiny subset of them can pass through the rigorous selection mechanisms, develop into mature follicles, and ovulate successfully [1]. Folliculogenesis is a complex and highly regulated process, mainly controlled by hormones, gene transcription, epigenetics, and other factors. Among these, histone modification in epigenetics plays a crucial role in oogenesis [2]. Histone modification can affect the affinity between histones and DNA duplexes, thereby altering chromatin compaction and relaxation during oocyte meiotic maturation [3]. Histone modification further modulates the recruitment of regulatory proteins (such as transcription factors) to chromatin and affects gene transcription during oogenesis and folliculogenesis [4]. Major types of histone modification include phosphorylation, methylation, ubiquitination, and acetylation. Among these, histone acetylation and deacetylation are indispensable for oocytes to accumulate and store requisite mRNAs, as well as for the epigenetic reprogramming of parental genomes during early embryonic development [5].

Histone acetylation is dynamically regulated by histone acetyltransferases (HATs) and histone deacetylases (HDACs) [6]. In mammals, 18 HDAC isoforms have been identified, which can be classified into four classes [7]. HDAC2 in class I HDACs exhibits high homology across species and is widely expressed in various tissues. Since HDAC2 lacks DNA-binding domains itself, it executes its biological functions by interacting with other transcription factors, forming homodimers, or assembling into multi-component repressor complexes [8]. Accumulating evidence indicates that HDAC2 may play a dominant role in oogenesis [9]. HDAC2 participates in establishing oocyte developmental competence from preantral to antral follicles in cattle [10]. HDAC2 contributes to establishing maternal genomic imprints and maintaining genomic integrity in oocytes in mice [11]. Moreover, HDAC2 plays an important role in regulating chromosome segregation and kinetochore function during oocyte maturation in mice [8]. HDAC2 triggers chromatin remodeling in ovarian granulosa cells through histone deacetylation [12]. Nevertheless, the stage-specific regulatory actions of HDAC2 across the full course of folliculogenesis remain poorly characterized, especially with respect to its functions in preantral follicle development.

Currently, studies on the role of HDAC2 in oogenesis mainly focus on single granulosa cells or oocytes. However, folliculogenesis is a complex process involving intercellular synergy between oocytes and granulosa cells. Studies focusing on isolated granulosa cells or oocytes cannot fully reflect the role of HDAC2 in the process of folliculogenesis. Moreover, the roles, mechanisms, and functional relationships of HDAC2 in distinct developmental stages of folliculogenesis have not been elaborated in detail. Given the core role of folliculogenesis in the fertility of livestock, this study uses an in vitro 3D culture system of mouse preantral follicles to investigate the regulatory role of HDAC2 in folliculogenesis and its underlying molecular mechanisms.

## 2. Materials and Methods

### 2.1. Chemicals and Animals

Minimum essential medium α (MEM-α, C12571500BT), ultra-low attachment 96-well plates (4520), and Leibovitz’s L-15 medium (11415064) were purchased from Thermo Fisher Scientific (Waltham, MA, USA). Fetal bovine serum (FBS, F8318), hyaluronidase (HAase, H3506), Insulin (5 mg/mL)–transferrin (5 mg/mL)–selenium (5 ng/mL) (ITS, 41400-045), and sodium pyruvate (PNa, P5280) were purchased from Sigma-Aldrich (St. Louis, MO, USA). Follicle-stimulating hormone (FSH, 1903112), human chorionic gonadotophin (hCG, 10-1255), and pregnant mare serum gonadotropin (PMSG) were purchased from Ningbo Second Hormone Factory (Ningbo, Zhejiang, China). Epidermal growth factor (EGF, AF-100-15-100) was purchased from PeproTech Inc. (Rocky Hill, NJ, USA). HDAC2 antibody (AF6470) was purchased from Afinily (Phoenix, AZ, USA). Santacruzamate A (SCA, HY-N0931) was purchased from MedChemExpress (MCE, Monmouth Junction, NJ, USA). Fluorescent secondary antibody (ab150077) and phalloidin–iFluor (ab176756) were purchased from Abcam (Cambridge, UK). Horseradish peroxidase (HRP, 7074) was purchased from Cell Signaling Technology (CST, Danvers, MA, USA). Immunohistochemistry kit (SA1020) and diaminobenzidine (AR1027-3) were purchased from BOSTER (Wuhan, Hubei, China). BCA protein quantification kit (KGP902) and total protein extraction kit (KGP2100) were purchased from KeyGEN (Nanjing, Jiangsu, China). Penicillin-streptomycin (for cell culture, C0222), Tubulin–Tracker Green (C1051S), MitoTracker Red (C1035), mitochondrial membrane potential (ΔΨm) assay kit (JC-1, C2003S), calcein acetoxymethyl ester (Calcein-AM, C2013S), propidium iodide (PI, ST1569), and 4′,6-diamidino-2-phenylindole (DAPI, C1005) were purchased from Beyotime (Shanghai, China).

Female ICR mice were obtained from the Comparative Medicine Center of Yangzhou University (Yangzhou, Jiangsu, China). Mice were kept under controlled temperature (23–25 °C) with a 12 h light/dark cycle. All animal procedures were performed in accordance with the guidelines of the Ethics Committee of Yangzhou University (License No. 202605016).

### 2.2. Collection of Mouse Ovaries

To investigate the expression of HDAC2 during folliculogenesis in mouse ovaries, the ovaries were harvested from female ICR mice at postnatal days 7, 10, 15, and 21, as well as those from 21-day-old female ICR mice after intraperitoneal injection of 5 IU PMSG for 12, 24, and 48 h, and PMSG for 48 h combined with 5 IU HCG for 12 h, respectively. Each group contained 5 female ICR mice.

### 2.3. Culture of Mouse Preantral Follicles In Vitro

Female ICR mice aged 12.5 days were euthanized with 5% isoflurane. Bilateral ovaries were promptly excised and transferred into L-15 medium (L-15, 10% FBS and 1% penicillin-streptomycin). Individual preantral follicles were mechanically dissected using a 0.3 × 13 RWLB needle under a stereomicroscope (SZ61-RT, Olympus, Tokyo, Japan). Preantral follicles with intact morphology and a diameter ranging from 100 μm to 130 μm were selected. A total of 400 morphologically intact preantral follicles were isolated for in vitro follicle culture across two independent biological replicates. For each replicate, 200 follicles were randomly assigned into five treatment groups, with 40 follicles per group. The selected follicles were cultured in ultra-low attachment 96-well plates defined as day 0 of culture, with each well containing 200 μL of follicle growth medium (MEM-α, 10% FBS, 1% ITS, 1% penicillin-streptomycin, 100 IU/L FSH, 0.33 mM PNa, and 50 mg/L vitamin C) and one follicle per well. Half-volume medium refreshment was performed every other day. On day 8, the medium was replaced with oocyte maturation medium (MEM-α, 1500 IU/L hCG and 10 μg/L EGF). Ovulation occurred and mature oocytes were obtained after 16 h of culture (on day 9).

### 2.4. Treatment of In Vitro Cultured Preantral Follicles with SCA

In vitro cultured follicles were treated with 5 μM SCA (a selective HDAC2 inhibitor) following four different treatment schedules. Method 1: Follicles were treated with SCA on culture day 0 for 2 days, followed by replacement with normal growth medium on day 2. Method 2: The follicles were treated with SCA on culture day 2 for 2 days, followed by replacement with normal growth medium on day 4. Method 3: The follicles were treated with SCA on culture day 4 for 2 days, followed by replacement with normal growth medium on day 6. Method 4: The follicles were treated with SCA on culture day 6 for 2 days, followed by replacement with normal growth medium on day 8.

### 2.5. Immunohistochemistry and Immunofluorescence Staining

Immunohistochemistry (IHC) and immunofluorescence (IF) staining were performed to localize HDAC2 in ovarian tissues. The ovaries were fixed in 3.7% formaldehyde for 24 h, dehydrated through a graded ethanol series, cleared in xylene, and embedded in paraffin. The sections (5 µm thick) were mounted onto glass slides and baked at 60 °C for 1 h. After dewaxing and rehydration, the samples were permeabilized with 0.1% Triton X-100 and incubated at 37 °C for 10 min. The slides were washed three times with PBS (pH 7.4) and pretreated with 3% H_2_O_2_ for 20 min to quench endogenous peroxidase activity. After washing with PBS, the samples were placed in citrate buffer (pH 6.0). Antigen retrieval was performed by treating the samples in a microwave oven at 92 °C for 5 min; the slides were then cooled and washed three times with PBS. Next, the sections were incubated with 5% BSA for 30 min at room temperature. After blocking, the sections were incubated overnight at 4 °C with an anti-HDAC2 primary antibody (1:200 dilutions). For IHC, the sections were incubated with an HRP-conjugated anti-rabbit secondary antibody for 1 h at room temperature, and DAB substrate was used for chromogenic development. For IF, the sections were incubated with anti-rabbit fluorescent secondary antibodies for 1 h and DAPI at a final concentration of 1 μg/mL for 15 min. Finally, the sections were dehydrated, mounted and imaged using a laser-scanning confocal microscope (CLSM, TCS SP8 STED; Wetzlar, Hessen, Germany).

### 2.6. Western Blot

Total proteins were extracted from ovarian tissues using the total protein extraction kit. Briefly, after incubation on ice for 10 min, the proteins were centrifuged at 12,000 r/min for 15 min. Protein concentration was determined using the BCA assay. Equal amounts of total proteins (20 µg) were loaded into each well and separated by electrophoresis in a 12% SDS-PAGE gel. The target proteins were electrotransferred onto polyvinylidene difluoride (PVDF) membranes via electroblotting (Millipore, Bedford, MA, USA). The membranes were blocked with 5% skim milk, followed by overnight incubation at 4 °C with anti-HDAC2 primary antibodies (1:1000 dilutions). After being washed with Tris-buffered saline containing Tween 20 (TBST), the membranes were incubated with a secondary antibody conjugated with HRP (1:5000 dilutions) for 1 h at room temperature. Immunoreactive bands were then visualized using an ultrasensitive ECL kit and a gel imaging system (Tannon Science & Technology, Shanghai, China). Band intensity was quantified with the software Quantity One (Version 4.6.2, Bio-Rad, Hercules, CA, USA). The experiments were performed independently in triplicate.

### 2.7. Assessment of Developmental Quality of In Vitro Cultured Follicles and Oocytes

On culture days 2, 4, 6, 8, and 9, the morphological structure of the follicles treated with SCA was observed and photographed under an inverted microscope (CKX53, Olympus). The rates of follicular survival, antrum formation, and oocyte extrusion were calculated after SCA treatment. On day 9, cumulus-oocyte complexes (COCs) were collected and treated with 0.1% hyaluronidase to digest cumulus cells, yielding denuded oocytes. The extrusion of the first polar body was observed under an inverted microscope, and the number of metaphase I (MI) and metaphase II (MII) oocytes was counted. Follicular diameters were measured using ImageJ software (Version 1.53t, NIH, Bethesda, MD, USA). Briefly, the maximum diameter of the follicle and the perpendicular diameter crossing the maximum axis were measured, and the average value of the two was taken.

### 2.8. Assessment of the Viability of In Vitro Cultured Follicles

To evaluate follicular viability, the cultured follicles were stained with Calcein-AM and PI in the dark on day 6, and the activity of follicles was observed using CLSM. Follicle survival was evaluated based on the fluorescence intensity of green and red signals emitted by ovarian granulosa cells and oocytes.

### 2.9. Observation of Microfilament Cytoskeletal Morphology and Distribution in Follicles

Follicles received SCA treatment starting on culture day 4 for a continuous 2-day period. On culture day 6, follicles were harvested and fixed with 3.7% formaldehyde for 15 min, followed by washes with PBS. Subsequently, the follicles were permeabilized with 0.1% Triton X-100 for 5 min and then incubated in Phalloidin-iFluor (1:1000 dilutions) for 1 h at room temperature in the dark. Next, the follicles were stained with DAPI for 20 min, mounted on glass slides, and imaged with CLSM.

### 2.10. Detection of Oocyte Spindle Morphology

Follicles received SCA treatment starting on culture day 4 for a continuous 2-day period. On culture day 8, the cultured follicles were induced to ovulate. Intracellular spindle morphology was labeled using Tubulin-Tracker Green. Briefly, approximately twenty oocytes were fixed with 3.7% formaldehyde for 15 min and then incubated in Tubulin-Tracker Green (1:1000 dilutions) for 1 h at room temperature in the dark. Next, the oocytes were stained with DAPI for 15 min, mounted on glass slides, and imaged with CLSM.

### 2.11. Detection of Mitochondrial Distribution in Oocytes

Follicles received SCA treatment starting on culture day 4 for a continuous 2-day period. On culture day 8, the cultured follicles were induced to ovulate. Intracellular mitochondrial distribution was labeled using MitoTracker Red. Briefly, approximately twenty oocytes were incubated in 100 nM MitoTracker Red (1:1000 dilutions) for 30 min at 37 °C in the dark. Next, the oocytes were stained with DAPI for 15 min, mounted on glass slides, and imaged with CLSM.

### 2.12. Measurement of ΔΨm in Oocytes

Follicles received SCA treatment starting on culture day 4 for a continuous 2-day period. On culture day 8, the cultured follicles were induced to ovulate. The ΔΨm in oocytes was detected using an enhanced ΔΨm assay kit, based on the relative ratio of red to green fluorescence. Briefly, approximately twenty oocytes were incubated in JC-1 (1:200 dilutions) for 30 min at 37 °C in the dark. Next, the oocytes were counterstained with DAPI for 15 min, mounted on glass slides, and imaged with CLSM.

### 2.13. Immunofluorescence Staining

Follicles received SCA treatment starting on culture day 4 for a continuous 2-day period. On culture day 8, the cultured follicles were induced to ovulate. Cumulus cells were removed, and denuded oocytes were collected. The oocytes were washed three times with PBS, fixed with 3.7% formaldehyde for 30 min at room temperature, permeabilized with 0.1% Triton X-100 for 10 min, and finally blocked with 5% bovine serum albumin (BSA) for 30 min. Subsequently, the oocytes were incubated with the corresponding primary antibody overnight at 4 °C. The next day, they were incubated with a fluorescent secondary antibody (1:200 dilution) for 1 h at room temperature. Finally, the oocytes were stained with DAPI for 15 min, mounted on slides after washing, and imaged with CLSM. Fluorescence intensity was analyzed with ImageJ software. Thirty oocytes were collected per group in each independent experiment, and the experiment was repeated three times independently.

### 2.14. RNA Isolation and Quantitative Real-Time PCR (qRT-PCR) Analysis

Total RNA was extracted from the ovaries and follicles using TRIzol (TaKaRa Bio, Inc., Dalian, Liaoning, China) according to the manufacturer’s instructions. Complementary DNA (cDNA) was synthesized using a PrimeScript RT Reagent Kit (TaKaRa Bio, Inc., Dalian, Liaoning, China). qRT-PCR was performed using the SYBR Premix Ex Taq II Kit (TaKaRa Bio, Inc., Dalian, Liaoning, China) according to the manufacturer’s protocols using a Roche LightCycler 480 II system. Levels of the target genes were normalized against GAPDH. The specific qPCR primer sequences are listed in Table 1. Relative mRNA abundance was calculated via the 2^−ΔΔCt^ method. Each gene was tested in triplicate, and all experiments were repeated in three independent biological replicates.

### 2.15. Statistical Analysis

All data are presented as mean ± standard deviation (SD). All experiments were performed in triplicate across three independent biological replicates. Statistical analysis was conducted with the SPSS software (Version 18.0, Chicago, IL, USA). Statistical significance of the differences was assessed by one-way ANOVA. Comparisons between control and treatment groups were performed using the LSD test. Statistical significance was indicated at * *p* < 0.05, ** *p* < 0.01, and *** *p* < 0.001 vs. control.

## 3. Results

### 3.1. Localization and Expression of HDAC2 in Mouse Ovary

**Figure 1:**
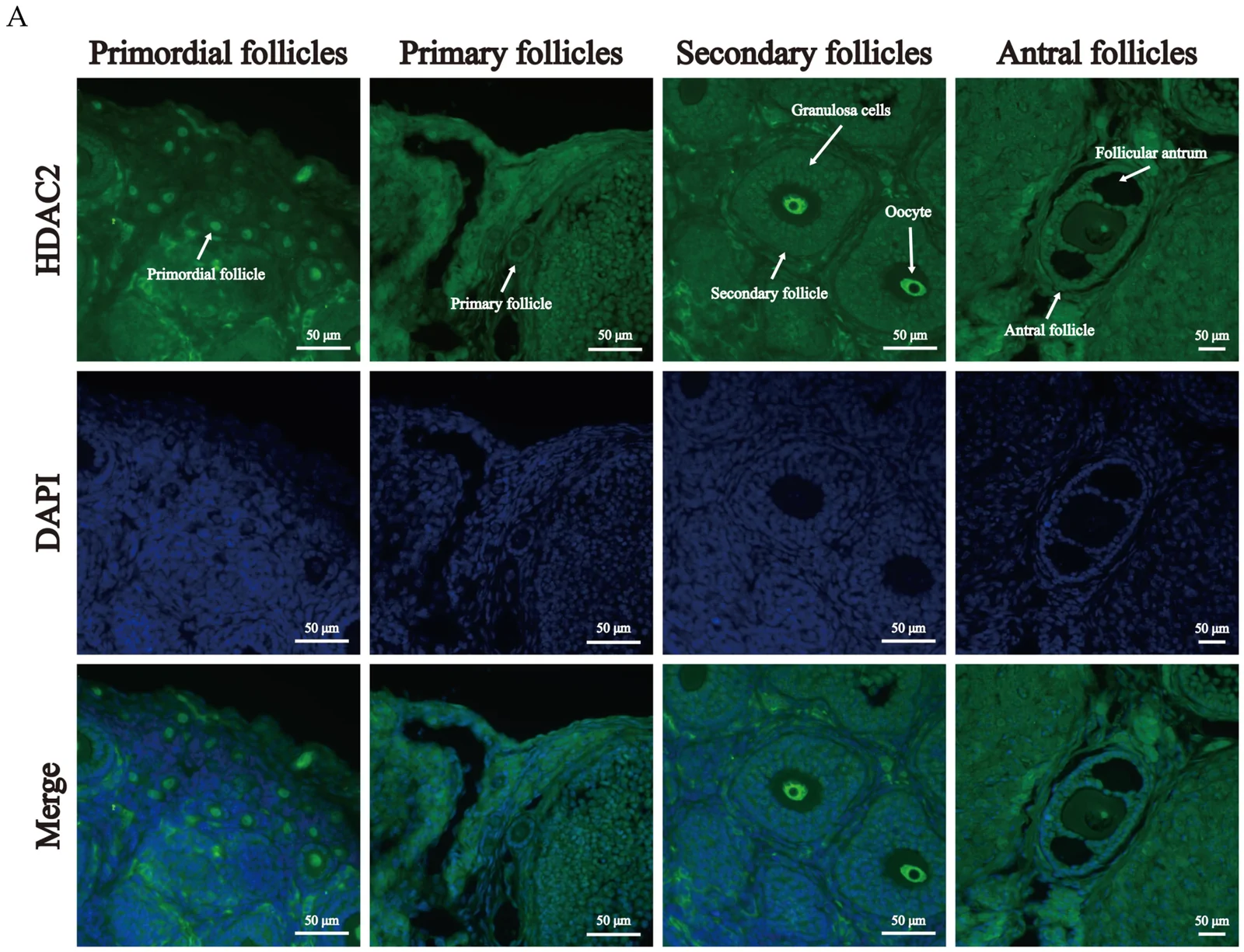

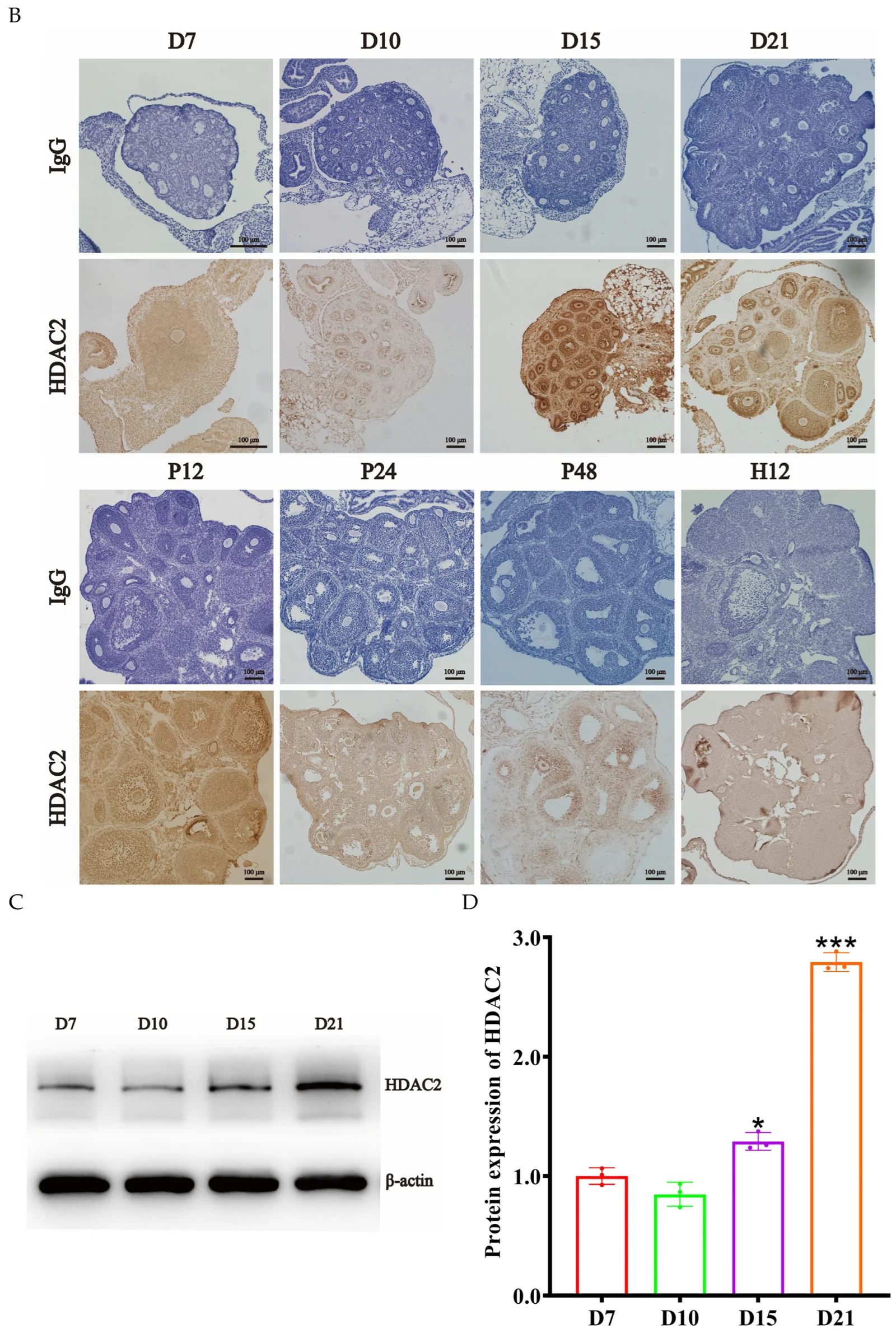

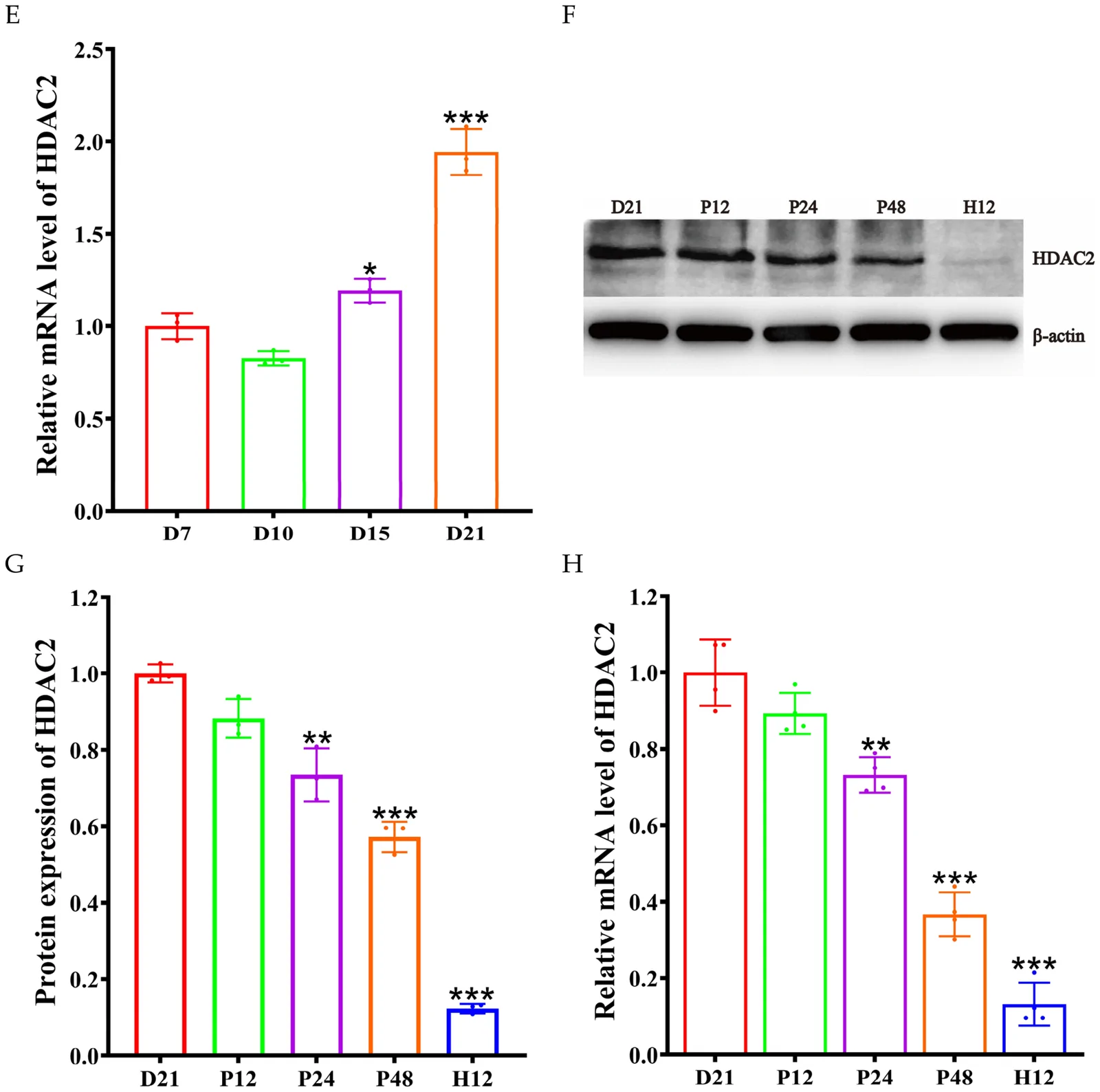
Localization and expression of HDAC2 in mouse ovary. (**A**) IF of HDAC2 in mouse ovaries. (**B**) IHC of HDAC2 in mouse ovaries at postnatal days 7 (D7), 10 (D10), 15 (D15), 21 (D21), and day 21 treated with PMSG for 12 (P12), 24 (P24), and 48 h (P48), and PMSG for 48 h and hCG for 12 h (H12), respectively. Scale bar, 50 μm. (**C**) Protein expression of HDAC2 in mouse ovaries at postnatal days D7, D10, D15, and D21, respectively. (**D**) Densitometric quantification of HDAC2. (**E**) Relative mRNA level of *HDAC2* in mouse ovaries at postnatal days D7, D10, D15, and D21, respectively. (**F**) Protein expression of HDAC2 in mouse ovaries at postnatal days P12, P24, P48, and H12. (**G**) Densitometric analysis of HDAC2. (**H**) Relative mRNA level of *HDAC2* in mouse ovaries of P12, P24, P48, and H12. * *p* < 0.05, ** *p* < 0.01, and *** *p* < 0.001 vs. D7 and D21, respectively.

IHC and IF were performed to localize HDAC2 in ovarian tissues. The results showed that HDAC2 was predominantly localized in the nuclei of ovarian granulosa cells and oocytes (Figure 1A,B). The protein and mRNA expression of HDAC2 were further determined by Western blot and qPCR (Figure 1C–H). Compared with postnatal day 7 mouse ovaries, HDAC2 expression had no significant difference at postnatal day 10, but was significantly increased at postnatal days 15 and 21 (Figure 1C–E). In addition, in 21-day-old mice, compared with the untreated control group, PMSG for 12 h did not significantly alter HDAC2 expression, while HDAC2 expression was significantly decreased following PMSG treatment for 24 and 48 h, and combined treatment of PMSG for 48 h plus hCG 12 h (Figure 1F,G).

### 3.2. In Vitro 3D Culture of Mouse Follicles

**Figure 2:**
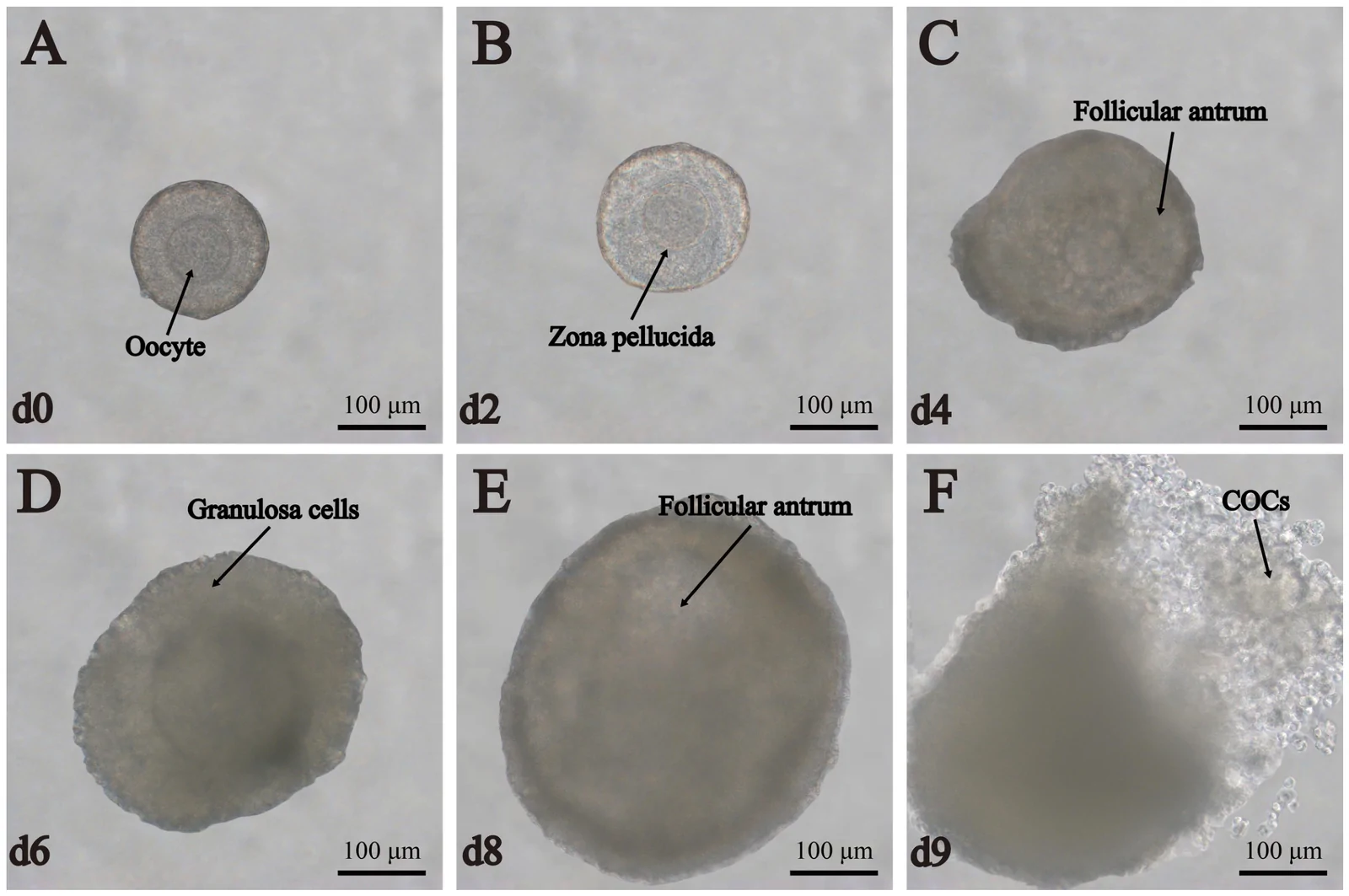
In vitro 3D culture of follicles. (**A**) Follicle on culture day 0 (d0). (**B**) Follicle on culture day 2 (d2). (**C**) Follicle on culture day 4 (d4). (**D**) Follicle on culture day 6 (d6). (**E**) Follicle on culture day 8 (d8). (**F**) Follicle on culture day 9 (d9). Scale bar, 100 μm.

Typical morphological characteristics were observed as preantral follicles developed into mature follicles during in vitro 3D culture. On culture day 0, the basement membrane had an intact structure, surrounded by 2–4 layers of granulosa cells, with an oocyte enclosed in the center that had an intact zona pellucida and uniform, translucent cytoplasm (Figure 2A). On culture day 2, the follicle diameter was significantly increased, whereas the oocyte diameter showed no significant change (Figure 2B). On culture day 4, antrum formation was observed (Figure 2C). On culture day 6, the follicular antrum further expanded (Figure 2D). On culture day 8, both the follicle and follicular antrum continued to enlarge, with follicle diameter reaching approximately 300 μm (Figure 2E). Furthermore, the extrusion of COCs was observed on day 9 (Figure 2F).

### 3.3. Expression of HDAC2 in Follicle and Oocyte of 3D Culture In Vitro

**Figure 3:**
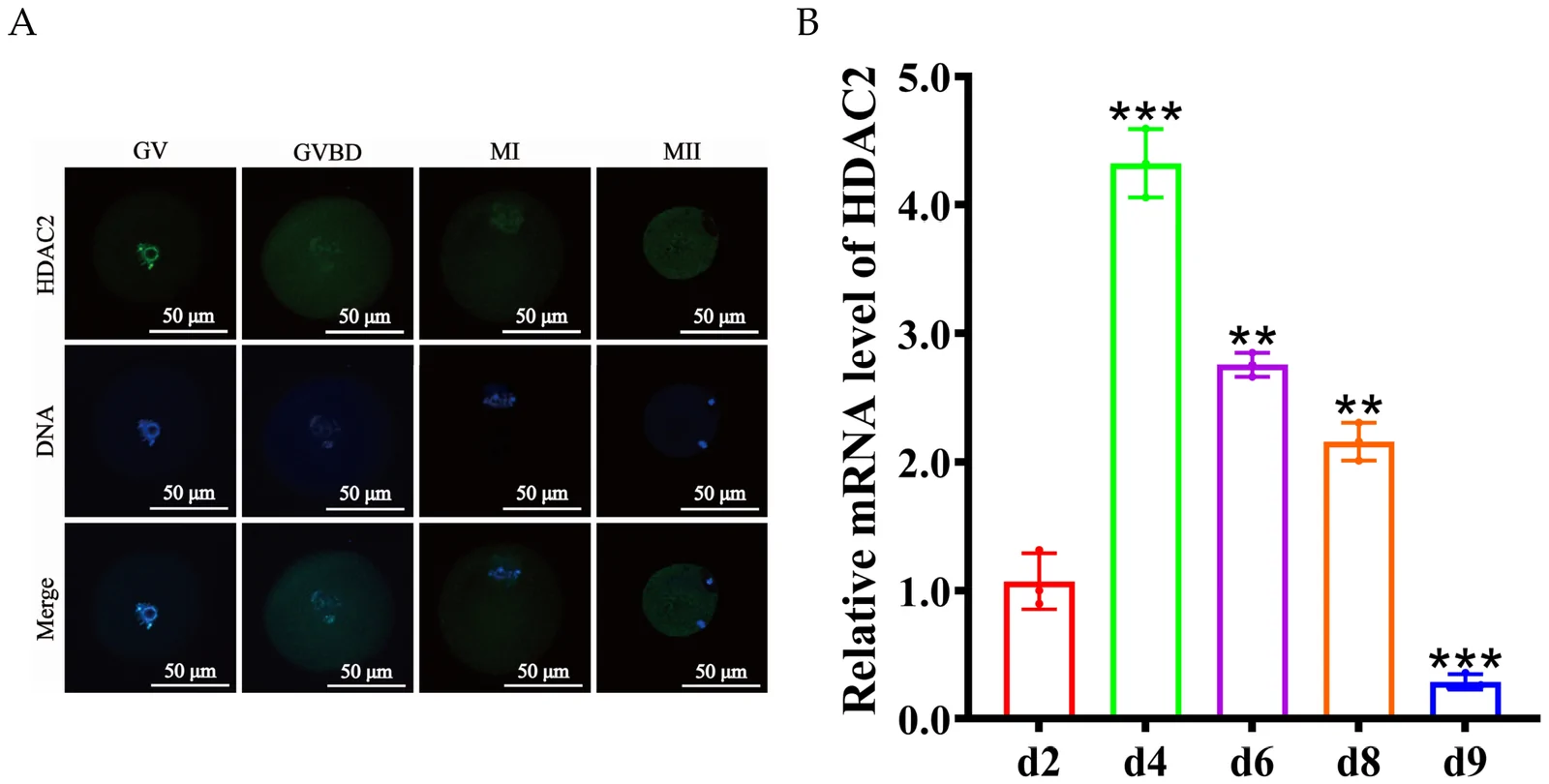
Localization and expression of HDAC2 in follicles and oocytes during in vitro 3D culture. (**A**) Representative IF images of HDAC2 in oocytes at GV, GVBD, MI, and MII stages. Scale bar, 50 μm. (**B**) Relative mRNA level of *HDAC2* in the follicles on culture days 2 (d2), 4 (d4), 6 (d6), 8 (d8), and 9 (d9). ** *p* < 0.01, and *** *p* < 0.001 vs. d2.

To explore the expression of HDAC2 in follicles and oocytes during in vitro 3D culture, the follicles were harvested on culture days 2, 4, 6, 8 and 9. IF results showed that HDAC2 was localized within the nuclei of the oocytes at GV, GVBD, MI, and MII stages, respectively (Figure 3A). qPCR results showed that the mRNA level of *HDAC2* was significantly increased on days 4, 6, and 8, peaking on day 4, while significantly decreasing on day 9 compared with day 2 (Figure 3B).

### 3.4. Effect of SCA on Follicular Morphology

**Figure 4:**
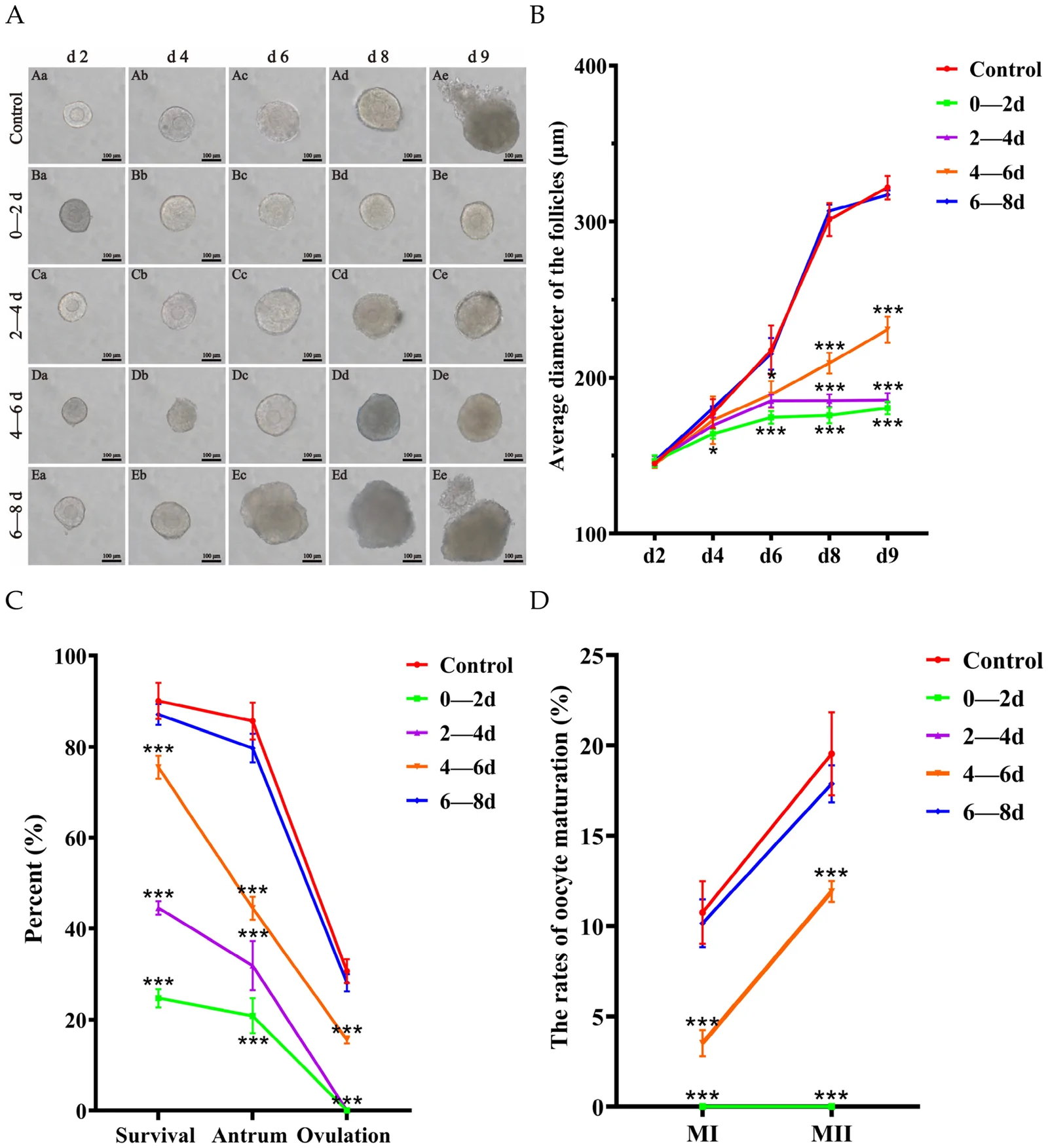
Effect of SCA on the growth and development of follicles during in vitro culture. (**A**) Representative images of follicular morphology on culture days 2 (d2), 4 (d4), 6 (d6), 8 (d8), and 9 (d9). (**B**) Quantitative analysis of follicular diameter. (**C**) Rates of follicular survival, antrum formation, and ovulation. (**D**) The proportions of oocytes at MI and MII stages. Scale bar, 100 μm. * *p* < 0.05 and *** *p* < 0.001 vs. Control.

To explore the effect of SCA on folliculogenesis, follicle diameter was smaller in the SCA group than that in the control group under SCA treatment from culture day 0 to day 2, day 2 to day 4, and day 4 to day 6, while there was no significant difference between day 6 and day 8 (Figure 4A,B). Consistently, the rates of follicular survival, antrum formation, and ovulation in the SCA group were lower than those in the control group under SCA treatment in the above three treatment intervals, and there were no significant differences between day 6 and day 8 (Figure 4C). Furthermore, the rates of MI and MII stage oocytes in the SCA group were lower than those in the control group under SCA treatment in the above three treatment intervals, and there were no significant differences between day 6 and day 8 (Figure 4D).

### 3.5. Effect of SCA on HDAC2 Expression and Panacetylation in Follicles and Oocytes

**Figure 5:**
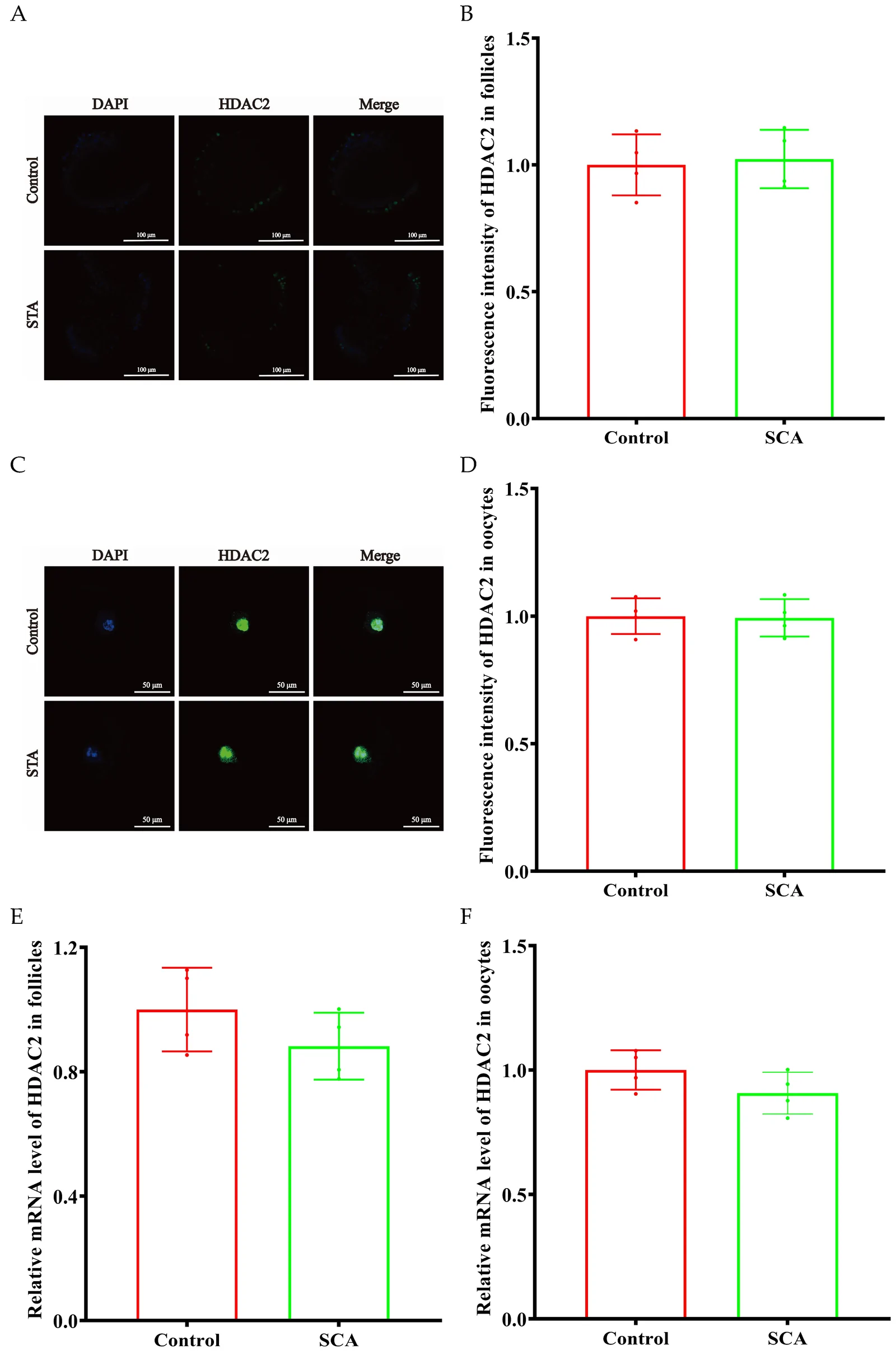

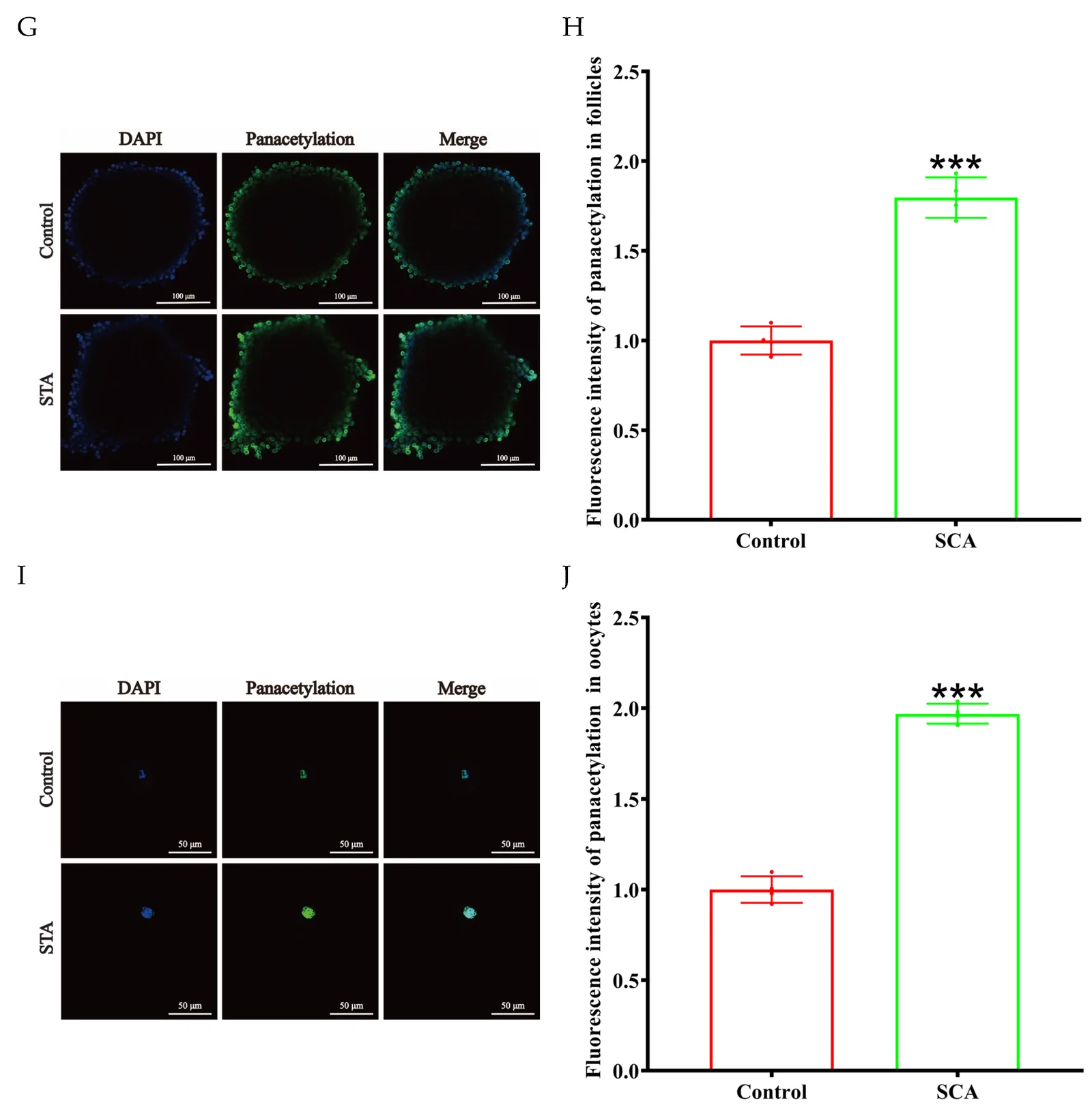
Effect of SCA on HDAC2 expression and panacetylation in follicles and oocytes during SCA treatment from day 4 to day 6. (**A**) Representative IF images of HDAC2 in follicles on day 6. Scale bar, 100 μm. (**B**) Quantitative analysis of HDAC2 fluorescence intensity in follicles on day 6. (**C**) Representative IF images of HDAC2 in oocytes on day 9. Scale bar, 50 μm. (**D**) Quantitative analysis of HDAC2 fluorescence intensity in oocytes on day 9. (**E**) Relative mRNA level of *HDAC2* in follicles on day 6. (**F**) Relative mRNA level of *HDAC2* in oocytes on day 9. (**G**) Representative images of panacetylation in follicles on day 6. Scale bar, 100 μm. (**H**) Quantitative analysis of panacetylation fluorescence intensity in follicles on day 6. (**I**) Representative images of panacetylation in oocytes on day 9. Scale bar, 50 μm. (**J**) Quantitative analysis of panacetylation fluorescence intensity in oocytes on day 9. *** *p* < 0.001 vs. Control.

To explore the effect of SCA on HDAC2 expression and panacetylation in follicles and oocytes, IF was performed in follicles on culture day 6 and oocytes on day 9. The results showed that there was no significant difference in fluorescence intensity of HDAC2 in follicles and oocytes between the SCA group and the control group (Figure 5A–D). Meanwhile, qPCR results were in accordance with those of IF (Figure 5E,F). In addition, the fluorescence intensity of panacetylation was significantly increased in the SCA group compared with the control group in both follicles and oocytes (Figure 5G,J).

### 3.6. Effect of SCA on Follicular Viability

**Figure 6:**
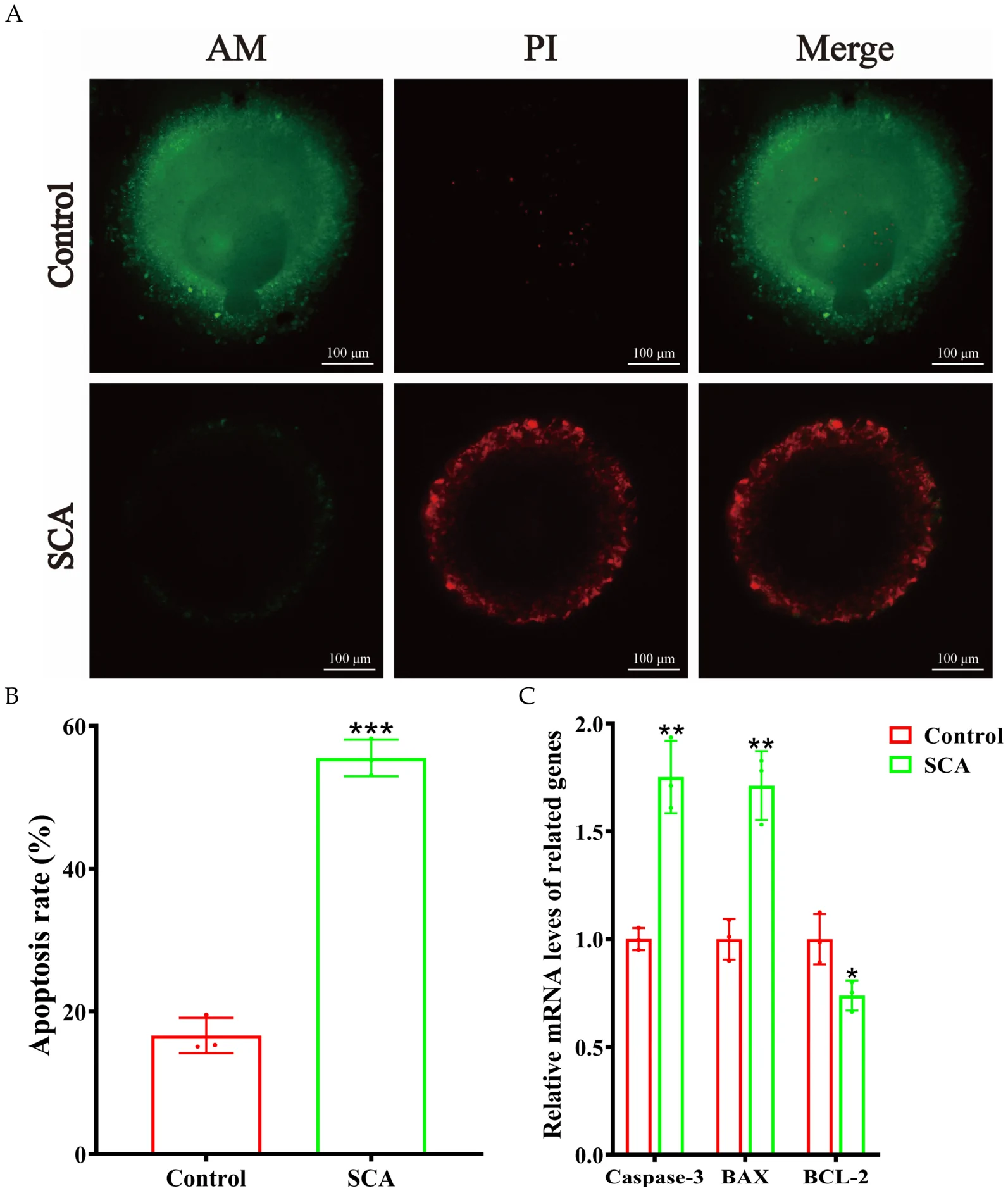
Effect of SCA on follicular viability under SCA treatment from day 4 to day 6. (**A**) Representative IF images of viability in follicles on day 6. AM labels the live cells (green); PI labels the apoptotic cells (red). Scale bar, 100 μm. (**B**) Rates of the apoptotic cells in follicles. (**C**) Relative mRNA levels of *Caspase-3*, *BAX*, and *BCL-2* in follicles on day 6. * *p* < 0.05, ** *p* < 0.01, and *** *p* < 0.001 vs. Control.

To evaluate follicular viability, Calcein–AM results showed that green fluorescence intensity of follicles was significantly decreased, while red fluorescence intensity was significantly increased in the SCA group compared with the control group on day 6 (Figure 6A). Additionally, the apoptosis rate of granulosa cells was significantly increased in the SCA group compared with the control group (Figure 6B). qPCR results showed that the mRNA levels of *BAX* and *Caspase-3* were significantly increased, while *BCL-2* was significantly decreased in the SCA group compared with the control group (Figure 6C).

### 3.7. Effect of SCA on the Morphology and Distribution of Microfilaments in Follicles

**Figure 7:**
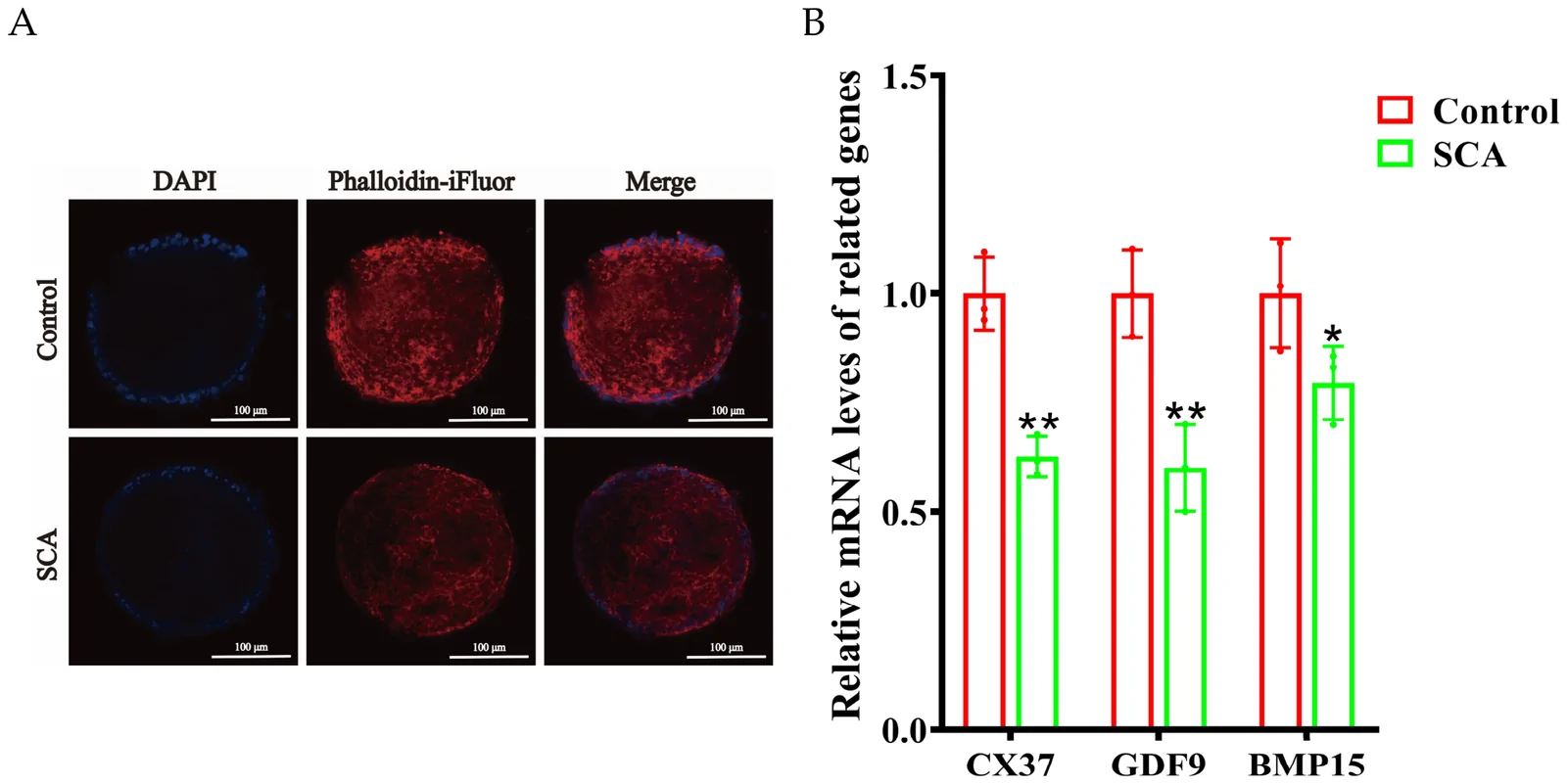
Effect of SCA on the microfilaments in follicles under SCA treatment from day 4 to day 6. (**A**) Representative images of phalloidin-iFluor staining in follicles on day 6. Scale bar, 100 μm. (**B**) Relative mRNA levels of *CX37*, *GDF9*, and *BMP15* in follicles on day 6. * *p* < 0.05 and ** *p* < 0.01 vs. Control.

To detect the effect of SCA on the morphology and distribution of microfilaments, phalloidin-iFluor staining results showed that actin filaments in the control group were predominantly distributed among granulosa cells with a relatively uniform reticular pattern and continuous microfilaments at intercellular junctions on day 6. In contrast, microfilaments were fragmented, unevenly distributed, and disruption at junction sites in the SCA group (Figure 7A). qPCR results showed that the mRNA levels of *CX37*, *GDF9*, and *BMP15* were significantly decreased in the SCA group compared with the control group (Figure 7B).

### 3.8. Effect of SCA on the Spindle Morphology and Chromosome Distribution in Oocytes

**Figure 8:**
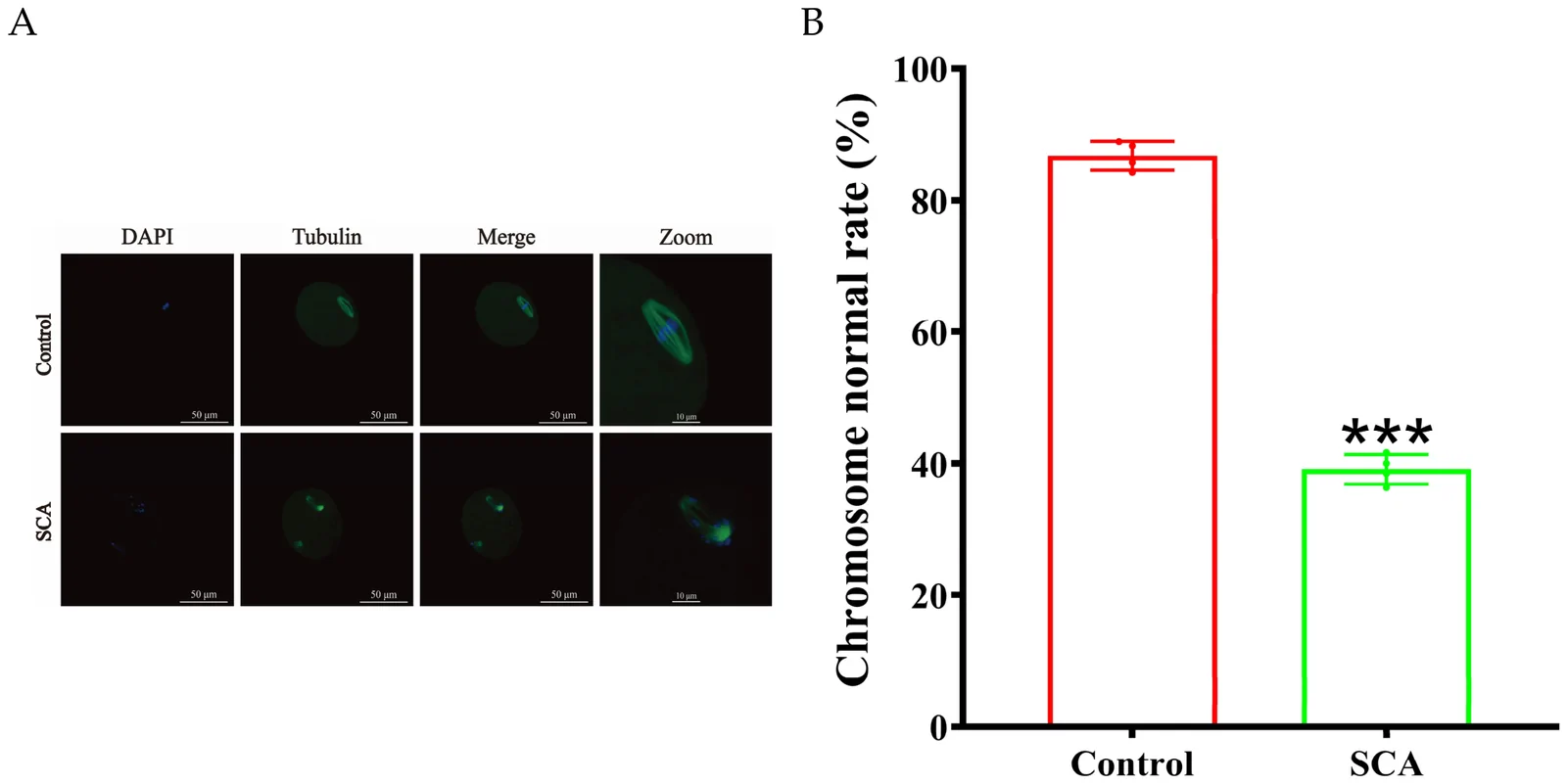
Effect of SCA on the morphology of the spindle in oocytes under SCA treatment from day 4 to day 6. (**A**) Representative images of the microtubules and chromosomes in oocytes by α-tubulin and DAPI staining on day 9. Scale bar, 50 μm. (**B**) Rate of normal chromosome in oocytes on day 9. *** *p* < 0.001 vs. Control.

To evaluate the effect of SCA on the spindle morphology and chromosome distribution in oocytes, Tubulin Tracker Green staining results showed that the chromosomes of oocytes were highly condensed and neatly aligned at the spindle equatorial plate in the control group and the spindle exhibited a fusiform shape with an intact structure and stable polarity (Figure 8A). However, the chromosomes on the spindle were mislocalized, with irregular alignment and defective condensation and segregation in the SCA group (Figure 8A). Moreover, the proportion of oocytes with normal chromosome arrangement was significantly decreased in the SCA group compared with the control group (Figure 8B).

### 3.9. Effects of SCA on Mitochondrial Distribution and Membrane Potential in Oocytes

**Figure 9:**
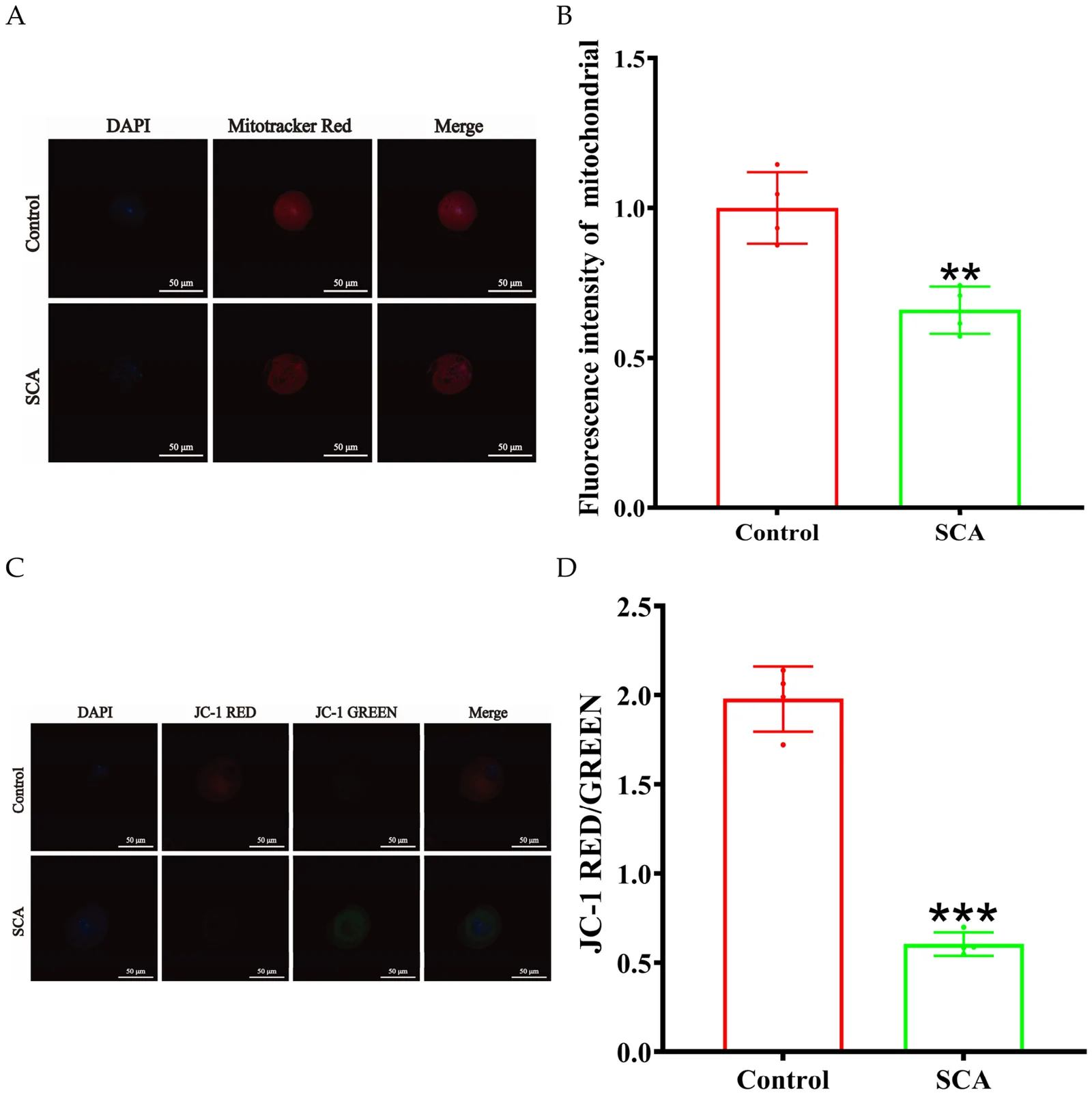
Effects of SCA on mitochondrial distribution and membrane potential in oocytes under SCA treatment from culture day 4 to day 6. (**A**) Representative images of mitochondrial distribution in oocytes on day 9. Scale bar, 50 μm. (**B**) Quantification of fluorescence intensity. (**C**) Determination of ΔΨm in oocytes on day 9. Scale bar, 50 μm. (**D**) The ratio of red to green fluorescence intensity. ** *p* < 0.01 and *** *p* < 0.001 vs. Control.

To evaluate the effect of SCA on the mitochondrial distribution and membrane potential in oocytes, MitoTracker Red staining results showed that mitochondria exhibited uniform cortical distribution in the control group, whereas they displayed disordered localization in the SCA group (Figure 9A). Fluorescence intensity had a significant decrease in the SCA group compared with the control group (Figure 9B). Moreover, JC-1 staining results showed that the red/green fluorescence ratio was significantly decreased in the SCA group compared with the control group (Figure 9C,D).

## 4. Discussion

HDACs are a class of epigenetic enzymes that exert critical functions in gene regulation [13]. Crosstalk between cellular metabolism and HDACs can modulate a wide range of biological processes. HDAC2 belongs to class I histone deacetylases and exhibits complex and diverse functions [14]. Beyond participating in gene transcriptional regulation, HDAC2 also plays key roles in multiple biological processes, such as DNA replication, DNA repair, and cell division [15]. In this study, we focused on the localization and expression of HDAC2 in mouse ovaries and the effects of HDAC2 inhibition on folliculogenesis. Through systematic investigations encompassing dynamic in vivo development, in vitro model validation, and molecular mechanism analysis, we uncovered the regulatory roles of HDAC2 in critical reproductive processes, including follicular growth and oocyte maturation. These findings provide an important experimental basis for understanding the epigenetic regulatory mechanisms governing folliculogenesis.

HDAC2 is expressed in the nuclei of both granulosa cells and oocytes. The nuclear localization of HDAC2 suggests that its core function lies in transcriptional regulation. As a member of the histone deacetylase family, HDAC2 compacts chromatin structure by removing histone acetyl groups, thereby repressing the transcription of target genes [9,16]. This nuclear localization is highly consistent with the requirement for spatiotemporally specific gene regulation during folliculogenesis. Granulosa cells rely on precise transcriptional regulation to achieve proliferation and differentiation, while oocytes need to maintain a specific gene expression profile at various stages of meiosis (from GV to MII stage) to ensure the orderly progression of their maturation process [17,18]. The nuclear localization of HDAC2 provides a structural basis for its involvement in the transcriptional regulation of these processes.

From the perspective of developmental stage-dependent expression, HDAC2 was gradually increased in the ovaries of 7–21-day-old mice, and this change was highly synchronized with the critical window of mouse ovarian development, the stage from the activation of preantral follicles to the formation of the follicular antrum [19,20]. The ovaries of 7-day-old mice mainly contain primordial and primary follicles, while those of 15–21-day-old mice gradually enter the transition stage from preantral to antral follicles. This process requires the orderly activation and silencing of a large number of genes, such as granulosa cell proliferation- and steroidogenesis-related genes [21]. The increased expression of HDAC2 may ensure the precise regulation of key folliculogenesis pathways by inhibiting the expression of redundant genes. After PMSG and hCG treatment, HDAC2 expression was significantly downregulated, further suggesting that its expression is regulated by endocrine signals. This stage-specific expression strongly indicates that HDAC2 may play a crucial regulatory role at key milestones of folliculogenesis, especially during antrum formation, an important morphological change process. This is consistent with previous studies concluding that HDAC2 is involved in key processes, such as the cell cycle, differentiation, and apoptosis [16,22,23]. FSH induces follicular growth, and LH triggers preovulatory maturation [24]. The downregulation of HDAC2 may release transcriptional suppression of ovulation-related genes, such as matrix metalloproteinases and downstream genes of the LH receptor, laying the foundation for the final maturation and ovulation of follicles.

The in vitro 3D follicle culture system established in this study recapitulated the core characteristics of in vivo folliculogenesis. Preantral follicles with intact structure (oocytes surrounded by 2–4 layers of granulosa cells) were observed on culture day 0, antrum formation initiated on day 4 marking the transition to the antral follicle stage, and COC extrusion was detected on day 9. This is consistent with the time course and morphological changes in folliculogenesis from preantral to mature ovulation in mice in vivo [25,26]. The reliability of this model was verified by the following two aspects. First, the follicular diameter increased continuously and stably during the culture period, which is consistent with the size characteristics of antral growth. Second, oocytes could successfully complete meiotic maturation from the GV stage to the MII stage. Taken together, a physiologically relevant in vitro experimental system was constructed for the subsequent evaluation of the regulatory effect of HDAC2.

In this model, HDAC2 expression was first increased and then decreased. HDAC2 reached a peak on day 4 (during antrum formation) and significantly decreased on day 9 (pre-ovulation). This change closely resembled the expression trend after gonadotropin treatment in vivo, further verifying the association between HDAC2 expression and key milestones of folliculogenesis. The peak expression on day 4 may be related to the gene regulatory requirements during antrum formation. Antrum formation relies on the coordination of granulosa cell-derived fluid secretion, extracellular matrix remodeling, and angiogenic signaling [27]. The high expression of HDAC2 may maintain the orderliness of antrum formation by inhibiting genes related to aberrant angiogenesis or excessive fluid secretion. The decrease on day 9 may release transcriptional suppression for the final maturation of oocytes, ensuring the smooth completion of meiosis.

HDACs exert essential functions in oocyte development. For instance, HDAC6 can disrupt the oocyte meiotic apparatus, thereby inhibiting early embryonic development [28]. In vivo experiments in mice have shown that the LH surge affects Amphiregulin (AREG) expression by downregulating HDAC3 expression, which in turn regulates oocyte maturation [29]. Additionally, trichostatin A (TSA) can alleviate smoking-induced ovarian tissue structure damage, inhibit the abnormal activation of HDAC1/2, restore the expression of autophagy-related proteins, and reduce the levels of pyroptosis-related proteins [30]. To better understand the role of HDAC2 in folliculogenesis, we investigated the response of follicles to exposure to SCA, a selective inhibitor of HDAC2, at different developmental stages. This study revealed that SCA affects folliculogenesis in a stage-specific manner. This phenomenon essentially reflects the differential functional necessity of HDAC2 at different stages of folliculogenesis. When SCA treatment was initiated on days 0–4, follicles were mainly in the preantral stage. SCA resulted in follicular growth arrest, antrum formation impairment, and eventual atresia, with no normal oocyte extrusion. This is because preantral follicles on days 0–2 are highly dependent on the transcriptional regulatory function of HDAC2 [10]. At this stage, follicles have not yet formed an antrum, and their development mainly relies on paracrine signals between oocytes and granulosa cells [31,32,33]. HDAC2 may maintain the balance of paracrine signals by inhibiting genes related to excessive apoptosis or abnormal proliferation of granulosa cells. When SCA inhibits the functions of HDAC2, this balance is disrupted, leading to increased granulosa cell apoptosis, disordered paracrine signals, and ultimately blocked basic development of follicles.

When SCA treatment was initiated on days 4–6, the follicles had entered the early antral follicle stage, which is characterized by the formation of an antrum filled with follicular fluid. There was no significant difference in follicular diameter at the initial stage of SCA treatment, but follicular growth was inhibited and atresia occurred from day 6 onwards. This phenomenon suggests that follicles in the early stage of antrum formation have a slightly higher tolerance to HDAC2 inhibition than those in the preantral stage. However, as development progresses, the role of HDAC2 in maintaining the survival and further growth of antral follicles gradually becomes prominent. With the accumulation of steroid hormones, proteins, and metabolites, direct transport of small molecules such as cAMP, Ca^2+^, and metabolic substrates glucose and amino acids can be achieved through intercellular communication between ovarian granulosa cells and oocytes [34]. Among them, the bidirectional transmission of cAMP can regulate the arrest and resumption of oocyte meiosis, while the supply of nutrients provides an energy basis for the growth and maturation of oocytes [35]. Meanwhile, oocytes also secrete factors such as GDF9 and BMP15 to reversely regulate the proliferation, differentiation, and steroidogenesis of granulosa cells [36,37]. This bidirectional intercellular communication constitutes folliculogenesis, which is a key molecular basis for ensuring the normal maturation of oocytes and the smooth development of follicles to ovulation. Sustained inhibition by SCA may impair these functions, thereby reducing oocyte quality and follicular atresia.

SCA treatment was applied on days 6–8, when follicles were at the late antral stage. Follicles developed normally and ovulated after SCA treatment. At this stage, follicles have completed most of their growth and genetic preparation, and inhibition of HDAC2 is insufficient to interfere with key preovulatory processes, such as the release of COCs and the completion of oocyte meiosis. Notably, SCA treatment initiated on day 6 had no significant impact, further confirming that the threshold requirement for HDAC2 activity decreases in the late stage of folliculogenesis.

SCA treatment did not alter the protein expression or mRNA transcription level of HDAC2 in oocytes, but markedly elevated the global lysine acetylation level. This verified that the regulation of acetylation status in oocytes by SCA was not achieved by downregulating HDAC2 expression, but by inhibiting its deacetylase activity [38,39]. As an important member of the class I histone deacetylase family, HDAC2 exerts a core deacetylation function during oocyte growth and meiotic maturation, and the stability of its expression and function is critical for maintaining the dynamic balance of chromatin structure [8]. Abnormally elevated global acetylation leads to excessive chromatin relaxation and reduced stability of nucleosome structure, which further interferes with the normal gene transcription regulatory program and affects the precise expression of key functional genes in oocytes [40]. A sustained and abnormally high acetylation status also disrupts chromosome condensation and segregation during oocyte meiosis, increases the risk of chromosome alignment defects and segregation errors, and ultimately reduces the maturation quality and developmental competence of oocytes [41]. SCA regulates acetylation modification in oocytes by inhibiting the enzymatic activity of HDAC2, which provides experimental evidence for understanding the mechanism underlying the maintenance of acetylation homeostasis during follicle development.

To further clarify the regulatory mechanism of HDAC2 in folliculogenesis, SCA induced granulosa cell apoptosis via increases in pro-apoptotic genes *BAX* and *Caspase-3* and a decrease in the anti-apoptotic gene *BCL-2*, indicating the critical role of HDAC2 in maintaining the survival of granulosa cells and oocytes. HDAC2 may sustain the survival balance of granulosa cells and oocytes by inhibiting the transcription of *BAX* and *Caspase-3*, or by activating *BCL-2* [42,43]. Granulosa cells serve as the nutritional support unit for folliculogenesis. Massive apoptosis of granulosa cells leads to insufficient nutrient supply to oocytes, disruption of signal transduction, and ultimately triggers follicular atresia [44]. This finding also validates the observations of arrested follicular growth and impaired ovulation after SCA treatment.

Microfilaments represent a core component of the cytoskeleton, which not only maintains the morphology and arrangement of granulosa cells but also participates in substance transport (e.g., cholesterol and nutrients) and signal transduction [45]. Within the follicular structure, the theca externa maintains morphological stability through a thick layer of microfilaments, while cytoplasmic microfilaments form dynamically changing microfilament lattice networks via aggregation at intersections [46]. Single-focused studies on either granulosa cells or oocytes alone struggle to accurately capture changes in microfilament-related physiological functions. This limitation arises because these simplified experimental systems lack the complete intercellular interaction network inherent in intact follicles [47,48]. In contrast, the 3D follicle culture model employed in this study enables direct observation of dynamic microfilament remodeling, which can be used as a functional indicator reflecting intercellular communication and substance transport efficiency.

The microfilament cytoskeleton was decreased and unevenly distributed after SCA treatment, indicating that SCA can affect the polymerization and distribution of follicular actin, and disrupt the normal structure and stable state of the microfilament cytoskeleton. As an important structural basis for maintaining follicular morphology, regulating intercellular connections and substance transport, microfilament remodeling directly affects the expression and function of gap junction proteins. In this study, with the decrease in microfilament content, the expression of CX37, a key molecule for communication between oocytes and granulosa cells in follicles, was significantly downregulated. CX37 is a key protein in oocyte-granulosa cell gap junctions, responsible for transmitting small molecule signals such as cAMP and IP3. Its downregulation blocks the communication between oocytes and granulosa cells, preventing oocytes from obtaining sufficient maturation signals, such as cAMP-regulated meiotic resumption [49,50,51]. At the same time, the expressions of oocyte-specific functional factors BMP15 and GDF9 were also significantly decreased, and their regulatory mechanisms may be related to two aspects. On the one hand, the impairment of CX37-mediated gap junction communication leads to insufficient nutrient and key signal molecule acquisition by oocytes, thereby inhibiting the transcription of their functional genes [52]. On the other hand, microfilament cytoskeleton disorder can directly affect intracellular mechanical signals and the activity of related pathways, further downregulating the expression of BMP15 and GDF9 [53,54]. GDF9 and BMP15 are growth factors secreted by oocytes that regulate the proliferation and differentiation of granulosa cells. Their downregulation further impairs granulosa cell function [55,56], ultimately leading to impaired coordination of folliculogenesis.

Oocyte maturation serves as a critical bridge connecting folliculogenesis and embryonic development, and its functional integrity is a prerequisite for ensuring successful female reproduction. This process involves a series of precisely regulated biological events, including meiotic resumption, chromosome assembly, and cytoplasmic maturation, which are accomplished through the coordinated actions of gene expression, signaling pathways, and intercellular communication [57]. Defects in oocyte maturation can directly lead to fertilization failure, reduced embryo quality, and even the development of reproductive disorders [58,59]. Early-stage and high-dose SCA treatments significantly reduced the proportion of MI/MII-stage oocytes, and this inhibitory effect exhibited distinct time- and dose-dependent characteristics. These indicate that inhibition of HDAC2 during the early stages of folliculogenesis may exert irreversible negative impacts on oocyte maturation.

The developmental competence of oocytes is directly correlated with their maturation status. Nuclear maturation involves the morphological characteristics and spatiotemporal dynamic remodeling of core nuclear structures such as chromatin and the spindle apparatus [57,60], while cytoplasmic maturation encompasses the functional remodeling of organelles and physiological and biochemical changes in the cytoplasmic matrix within the cytoplasm [61]. Extensive research has confirmed that oocytes can only successfully fertilize and maintain the normal developmental competence of embryos when the nuclear and cytoplasmic maturation processes coordinately proceed and are ultimately completed [62]. In the present study, SCA induced abnormal spindle structure and disordered chromosome alignment in the MII-stage oocytes. The spindle apparatus is a core structure responsible for chromosome segregation during meiosis, and its integrity relies on the precise regulation of genes associated with microtubule assembly and spindle pole positioning [63]. Following HDAC2 inhibition, excessive histone acetylation may lead to aberrant expression of core checkpoint complex genes, or directly affect the deacetylation modifications of non-histone substrates such as microtubule-associated proteins, thereby disrupting spindle assembly and chromosome condensation [64,65]. Disordered chromosome alignment can induce meiotic errors and aneuploidy. Even if some oocytes reach the MII stage, their subsequent fertilization and embryonic developmental capacity may be significantly impaired. This finding provides a molecular basis for the reduced embryonic developmental competence observed after SCA treatment.

As the most abundant and functionally critical organelles in oocytes, mitochondria directly determine the maturation quality and developmental competence of oocytes [66]. During oocyte maturation, mitochondria undergo significant dynamic remodeling. Mitochondria disperse small clusters uniformly in the GV stage, and gradually migrate to the cortical region during the MI and MII stages, forming a polarized distribution pattern surrounding the spindle apparatus. This spatial rearrangement ensures the precise supply of energy during meiosis [67,68]. Concurrently, mitochondrial function undergoes synchronous regulation. By upregulating the activity of oxidative phosphorylation-related enzymes, mitochondria enhance ATP production efficiency, providing energy support for key nuclear and cytoplasmic maturation events such as chromatin condensation, spindle assembly, and cortical granule migration [66,69]. This study reported the negative effects of HDAC2 inhibition on oocyte mitochondria. SCA induced abnormal mitochondrial distribution, characterized by aggregation into clusters or diffuse uneven distribution, accompanied by changes in ΔΨm. Mitochondria serve as the cell’s energy factory, and their normal reticular distribution and membrane potential are crucial for providing ATP required for oocyte maturation, maintaining calcium homeostasis, and regulating apoptosis [70,71]. Existing studies have confirmed that insufficient mitochondrial DNA copy number, reduced membrane potential, or imbalanced mitochondrial dynamics are closely associated with decreased oocyte fertilization rates and embryonic developmental arrest [72], fully demonstrating the core regulatory role of mitochondria during oocyte maturation. Dysregulation of mitochondrial function is likely an important factor contributing to low follicular developmental competence and oocyte maturation failure.

The core value of this study lies in clarifying the stage-specific expression pattern of HDAC2 during mouse folliculogenesis and revealing its multifaceted roles as an epigenetic regulator in follicular growth, oocyte maturation, and steroidogenesis. Through intervention experiments using SCA, a specific inhibitor of HDAC2, the functional requirement for HDAC2 is dependent on developmental stage and dosage, providing new insights into understanding the epigenetic regulatory network of folliculogenesis. The established correlation between the in vitro 3D follicle culture model and the dynamic expression of HDAC2 offers a reliable experimental platform for future investigations into the epigenetic regulation of folliculogenesis. However, this study has certain limitations. The downstream effector molecules regulated by HDAC2 were not identified through transcriptomic analysis. Future studies should combine in vivo experiments, gene editing technologies, and transcriptome sequencing analysis to further refine the regulatory mechanism of HDAC2 in folliculogenesis, thereby providing more robust evidence for its application in reproductive biotechnology.

## 5. Conclusions

In summary, HDAC2 participates in the transcriptional regulation of mouse folliculogenesis through nuclear localization. Its expression dynamically changes with ovarian developmental stages and gonadotropin treatment, and exhibits an initial-increase-followed-by-a-decrease pattern synchronized with key developmental milestones in 3D-cultured follicles in vitro. SCA exerts stage-dependent regulatory effects on folliculogenesis. Follicles are more sensitive to SCA in the early stage of development, while showing a certain degree of tolerance in the late stage. The underlying mechanisms involve promoting granulosa cell apoptosis, disturbing oocyte meiosis, and disrupting microfilament architecture and intercellular communication. This study not only expands our understanding of epigenetic regulation governing folliculogenesis but also provides a new target and an experimental basis for the optimization of in vitro follicle culture and the regulation of reproductive performance.

## Figures and Tables

**Table 1:** The sequences of primers used for qPCR.

Gene	GeneBank No.	Forward (5ʹ–3ʹ)	Reverse (5ʹ–3ʹ)	Product (bp)
*GAPDH*	NM_001289726.2	TGTGTCCGTCGTGGATCTGA	TTGCTGTTGAAGTCGCAGGAG	150
*HDAC2*	NM_008229.2	GATGGAGAAGACCCGGACAA	TGGGGTCTGTTTTCTCACCA	241
*BCL-2*	NM_009741.5	GATGACTGAGTACCTGAACCG	CAGAGACAGCCAGGAGAAATC	149
*Caspase-3*	NM_001284409.1	TGACTGGAAAGCCGAAACTC	GCAAGCCATCTCCTCATCAG	101
*BAX*	NM_001411994.1	GCTTCAGGGTTTCATCCAGGATCG	ACTCGCTCAACTTCTTGGTAGATGC	283
*CX37*	NM_008120.3	CCCACATCCGATACTGGGTG	CGAAGACGACCGTCCTCTG	220
*GDF-9*	NM_001431151.2	GATGGTGGACCTGCTGTTTA	GAGGAAGAGGCAGAGTTGTTC	124
*BMP-15*	NM_009757.5	AGTGTACCTCAGCCTTCCT	GGGCAATCATACCCTCATACTC	179

## Data Availability

The data that support the findings of this study are available upon request.
